# Hybrid neural network method for damage localization in structural health monitoring

**DOI:** 10.1038/s41598-025-92396-9

**Published:** 2025-03-07

**Authors:** Fatahlla Moreh, Yusuf Hasan, Zarghaam Haider Rizvi, Sven Tomforde, Frank Wuttke

**Affiliations:** 1 Geomechanics and Geotechnics, Kiel University, Kiel, 24118 Germany; 2 Computer Engineering, Aligarh Muslim University, Aligarh, India; 3 Department of Civil and Environmental Engineering, University of Waterloo, Waterloo, Canada; 4 Institute of Computer Science, Kiel University, Kiel, 24118 Germany

**Keywords:** Engineering, Materials science, Mathematics and computing, Physics

## Abstract

The detection of cracks in large structures is of critical importance, as such damage can result not only in significant financial costs but also pose serious risks to public safety. Many existing methods for crack detection rely on deep learning algorithms or traditional approaches that typically use image data. In this study, however, we explore an innovative approach based on numerical data, which is characterized by greater cost efficiency and offers intriguing research implications. This study emphasizes the evaluation of hybrid RNN-CNN models in comparison to the pure CNN models previously utilized in related research. Our proposed model incorporates a single RNN layer, complemented by essential supporting layers, which contributes to a reduction in complexity and a decrease in the number of parameters. This design choice results in a more streamlined and efficient architecture. Our experimental results reveal an accuracy of 78.9%, which, while slightly lower than the performance of conventional CNN models, underscores the potential of RNN layers in crack detection tasks. Importantly, this work demonstrates that integrating additional RNN layers can effectively enhance crack detection capabilities, particularly given the significance of preserving spatial information for accurate crack segmentation. These findings open avenues for further exploration and optimization of RNN-based methods in structural damage analysis, suggesting that the strategic use of RNNs can complement CNN models to achieve robust performance in this domain.

## Introduction

The rapid advancement of Artificial Intelligence (AI) is reaching new heights in many fields and raises hopes of applying advanced models to enhance human safety. A promising application area is Structural Health Monitoring (SHM), which aims to continuously monitor large structures and constructions to detect potential hazards early. Structures such as bridges, airplanes, tunnels, and high-rise buildings pose significant risks to human life due to material fatigue or cracks. Even small cracks can alter material behavior, weaken structural integrity, and, in extreme cases, lead to total collapse, resulting in substantial losses of both property and lives.

The main aim of structural health monitoring is to assess the condition of a structure using ultrasonic sensor data and to make informed decisions regarding maintenance or decommissioning based on this information. Advances in SHM research highlight the increasing demand for data processing and analysis methods across various levels of SHM task. AI-based approaches, especially deep learning (DL) models, show great promise in this domain^1^, as they allow models to automatically learn task-specific features without relying on feature extraction and high-level domain knowledge.

A significant advantage of Deep Learning models is that they are able to develop hierarchical representations directly from raw data without any feature selection process. Through layered neural networks and non-linear transformations, deep neural networks (DNNs)^2^ learn complex relationships and capture a broad range of structural properties.

Alongside image-based approaches, wave propagation has also been explored for damage detection. Nature of training data plays a critical role in selecting suitable network architectures. Dense networks, one-dimensional Convolutional Neural Networks (1D-CNNs), Recurrent Neural Networks (RNNs), Gated Recurrent Units(GRUs), and Long Short-Term Memory (LSTM) networks are commonly used to analyze vibration signals in the time domain, while two-dimensional CNNs are well-suited for learning features in the frequency domain or from sensor arrays.

When waves interact with cracks, the wavefield deviated from its original path; as the wavefront encounters a damaged surface, reflections and refractions occur. For crack localization, this interaction is utilized by using sensors that detect these deflections,

In the previous work^3^, 1-D version of Densenet backbone was used in the encoder to extract the temporal features from the data. In MicroCrackRNNNet, the Temporal encoder with GRU units extracts crack-relevant features from the data, while the decoder maps the damaged areas according to these features.

In contrast, the present study represents the first attempt to employ a hybrid RNN-CNN model for this problem. The results demonstrate that crack detection is not limited to solely using CNN or RNN models but can be effectively enhanced through a combined model. A major advantage of the hybrid RNN-CNN approach is its ability to leverage the strengths of both model types: the RNN’s capability for handling sequential data and temporal dependencies, alongside the CNN’s powerful feature extraction.

This hybrid approach benefits from a reduced number of parameters compared to more complex standalone CNNs, which significantly lowers computational costs and model complexity. The efficient architecture opens up new avenues for training more sophisticated models while maintaining manageable resource requirements. This efficiency is particularly beneficial for Structural Health Monitoring (SHM), where large and complex datasets are common, and computational resources can be a limiting factor.

The hybrid model is particularly well-suited for our data structure, which comprises temporally segmented sequences where critical changes in wave characteristics are observed at specific time intervals. The RNN component allows MicroCrackRNNNet to accurately capture these temporal differences and dependencies, while the CNN layers ensure robust feature extraction from the data. This synergy results in a more comprehensive training process that enhances the detection of cracks and other structural anomalies.

Comparative analysis between CNN, RNN, and the hybrid RNN-CNN models provides valuable insights into which model is better suited for specific SHM tasks. The results from this study contribute to the broader body of SHM research by demonstrating the potential of hybrid neural network model and expanding the toolkit available for advanced structural analysis.

## Related work

The Damage Detection problem is comparable to semantic segmentation in Deep learning, In the previous studies^3^ Densenet model with 1D CNN^4^ were used to extract temporal features from the wavefield data. However 1D CNNs require a large amount of labeled data to effectively train the model and achieve good parameter convergence. For domains with limited annotated data, this dependency can lead to suboptimal performance and overfitting^5^, even with data augmentation techniques. Noise in time series data, particularly from high-frequency perturbations, interferes with feature extraction. Techniques like Fast Fourier Transform (FFT)^6^ have been employed to convert temporal signals to the frequency domain to reduce noise, but this approach may not fully resolve the problem, especially for non-stationary signals. 1D CNNs may struggle to capture multiscale patterns within time series data. Approaches involving multiple branches^7,8^ with varied filter sizes or an adaptive filter in the first convolutional layer attempt to address this, but they add complexity and require additional computation. Fixed filter sizes or inappropriate receptive fields can miss critical patterns and fluctuations in the data.

While transfer learning with limited or full fine-tuning offers some improvements, these methods require careful selection of source domains. When data similarity is low, performance can degrade, limiting the generalizability of CNNs across domains. High parameter counts in some CNN configurations make these models computationally intensive and unsuitable for mobile or resource-constrained devices, contrasting with lighter alternatives like transfer learning-based SVM approaches^9^.

RNNs^10^ are the most often utilised NN models for time series data. They are particularly popular in natural language processing^11–13^. RNNs, like ANNs, have universal approximation capabilities. Unlike ANNs, recurrent cells’ feedback loops consider both sequence order and temporal dependency.

RNNs have an internal state that updates with each time step, allowing them to detect sequential dependencies. They use feedback loops to connect neurones to previous outputs, forming a “memory” over time, which is required for processing time-series data. The circular connections between neurones enable RNNs to retain information from prior time steps and apply it to the present time step, making them useful for temporal patterns. RNNs use methods such as Backpropagation Through Time (BPTT)^14^ and Real-Time Recurrent Learning (RTRL)^11^ to update weights based on temporal relationships within the sequence, but BPTT is more widely used due to its computational efficiency. An RNN model is made up of multiple RNN units. Among the most widely used units for sequence modeling tasks are the Elman RNN cell^15^, LSTM cell^16^, and GRU^16^.

In^17^ the authors employ a two-level LSTM In the first stage, they used an LSTM-based noise model to identify and filter out known noise signals, effectively reducing interference. At the second level, another LSTM model is trained to detect crack signals by filtering out any remaining, unknown noise from the denoised signal of the first stage. This layered approach allows for effective noise mitigation and improves the accuracy of crack detection in a real railway environment, demonstrating the LSTM network’s suitability for managing both short- and long-term dependencies in complex time series data.

Wang et al.^18^ investigate defect depth determination within carbon fiber reinforced polymers (CFRP) using a LSTM model trained on laser infrared thermography (LIT) data. Compared to traditional recurrent and convolutional neural network methods, the LSTM network demonstrated superior accuracy in distinguishing defect depths based on temperature decay characteristics. The study leverages thermographic signal reconstruction (TSR) to preprocess raw thermal signals, effectively enhancing data quality by reducing background noise. This preprocessing approach, coupled with the LSTM’s ability to capture both short- and long-term temporal dependencies, allows for precise depth estimation in CFRP materials, achieving recall rates above 95 In the study^19^ uses a GRU (Gated Recurrent Unit) neural network to address real-time crack damage detection on box girders. The GRU model excels over traditional methods in accurately detecting crack location and length, showing high resistance to noise and robust approximate prediction for cracks outside the training set. This study demonstrates GRU’s effectiveness in processing temporal data for structural health monitoring and crack detection.

### Dataset facts

The dataset used to train the model is generated for the validated lattice element model developed for the detection of damage in cemented granular media. The previous study^20,21^ provides details of the model and the advantages of lattice modeling and its precision for such complex crack wave and crack-crack interaction problems with dynamic and impact loading.

The materials in the simulation are represented by Lattice Element Models (LEMs)^20,21^, where the nodes correspond to the unit cell centers connected by beams that can endure normal, shear, and bending forces. The system’s strain energy is minimized during the simulation, leading to the removal or stiffness reduction of elements that exceed a specific energy threshold, thus simulating cracking. This approach uses Voronoi cells and Delaunay triangulation to generate the lattice elements, facilitating mesh-independent computations of failure response. The dynamic lattice-element method efficiently produces large volumes of data suitable for training deep neural networks, offering a viable alternative to costly real-world sample generation.

The dataset consists of synthetic material samples, each having a single crack with random properties, including length, position, and orientation. A small subset of non-cracked samples is also included for comparison. Despite the random nature of the sample generation process, care was taken to ensure that the crack properties-size, position, and orientation-follow an independent and identically distributed pattern.

The validated and verified model is then used to produce a large dataset with different crack sizes, locations, orientations, azimuths, and thicknesses of the cracks in the heterogeneous granular cemented media. The material generated as its representative element volume is relevant and similar to cement concrete, rocks, fragmented cemented solids, and functionally graded concrete. Therefore, the deep learning model developed could solve the problem of crack identification within such complex structures, which is very subjective and cumbersome with the present forward and inverse identification problems due to its limitation with granular solids with porous structures.

The wavefield generated in each simulation is crucial for detecting cracks, as the interaction between the wave and any cracks causes changes in the wave pattern, which can be analyzed using deep learning techniques.

The experimental study for selected cases is presented, and the Boundary Element Method is also used to validate the lattice element method model.

For model training, low-resolution binary images (e.g., 100x100 pixels) are generated for localization of cracks, with the possibility to adjust resolution to refine model predictions. The model was trained and tested on normalized datasets as shown in paper^3^, comprising 4,810 samples for training, 910 for validation, and 1,760 for testing.

**Fig. 1:**
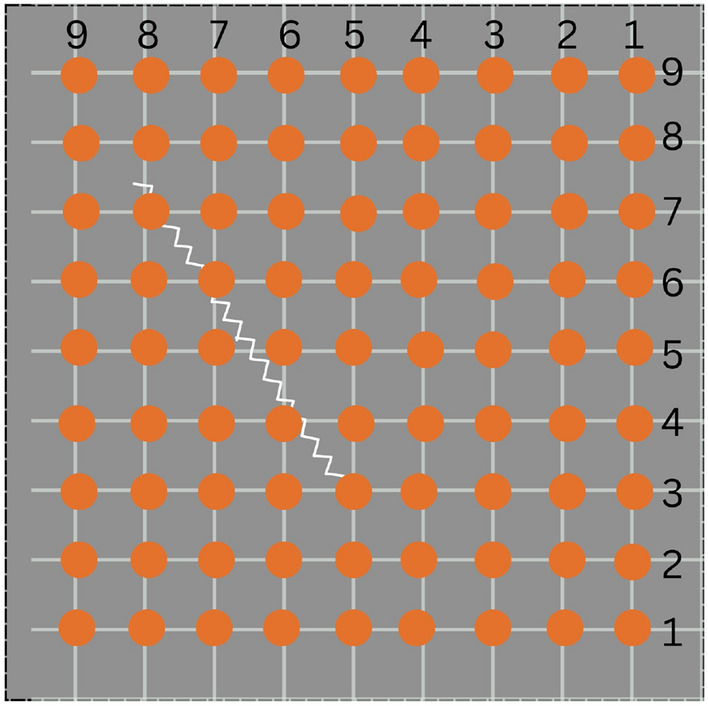
The placement of sensor array in sample domain.

Figure 1 shows the placement of sensors in a sample domain. There are a total of 81 sensors (a grid of 9x9) that generate a wavefield of shape 2000x9x9x2. For each sample, a seismic wave is initiated from the middle point of the edge of the plate by a force of 1000N, and the resulting displacements in both x- and y-directions are recorded by a grid of sensors over 2000 timesteps.

The dataset used for training does not have physical sensors. Instead, the sensors referenced here are numerical data collection points in the domain. The lattice element method discretizes the plate structure with over 300,000 points. 80 points from the point cloud are selected to observe the behavior of displacement with time due to crack-crack and crack-wave interaction problems inside the structure. Physically placing 80 sensors in a tight space would be a challenging task.

The numerical data undergoes min-max normalization to expedite neural network training, scaling is an effective preprocessing technique that enhances the network’s learning capabilities. With the data scaled appropriately, the signals are fed into the neural network, ensuring accurate and reliable learning without introducing unnecessary computational strain. Scaling the values between -1 and 1. This helps the model’s layers focus on learning features rather than normalizing the data, ultimately boosting the model’s efficiency.

This study, based on the Dynamic Lattice Element Method, can be adapted for real-world applications through a systematic approach that bridges numerical simulations with experimental validations. Initially, experiments using ultrasonic or acoustic wave propagation techniques on actual structures will allow for direct comparisons between measured and simulated wave signals, thereby confirming the model’s accuracy. Material parameters, such as Young’s modulus and density, will be carefully calibrated to reflect the true behavior of structural materials, accounting for heterogeneity and environmental influences. Furthermore, integrating sensor networks-such as piezoelectric transducers-into real structures will enable the continuous collection of wave propagation data, which the deep learning model can then analyze for crack detection. The method’s versatility will be demonstrated by testing across various materials (e.g., concrete, steel, composites) and crack patterns, ensuring its general applicability. Finally, optimizing the computational efficiency of the model will pave the way for its implementation in real-time structural health monitoring (SHM) systems for bridges, buildings, aircraft, pipelines, and other critical infrastructure.

## Deep neural networks for crack detection

**Fig. 2:**
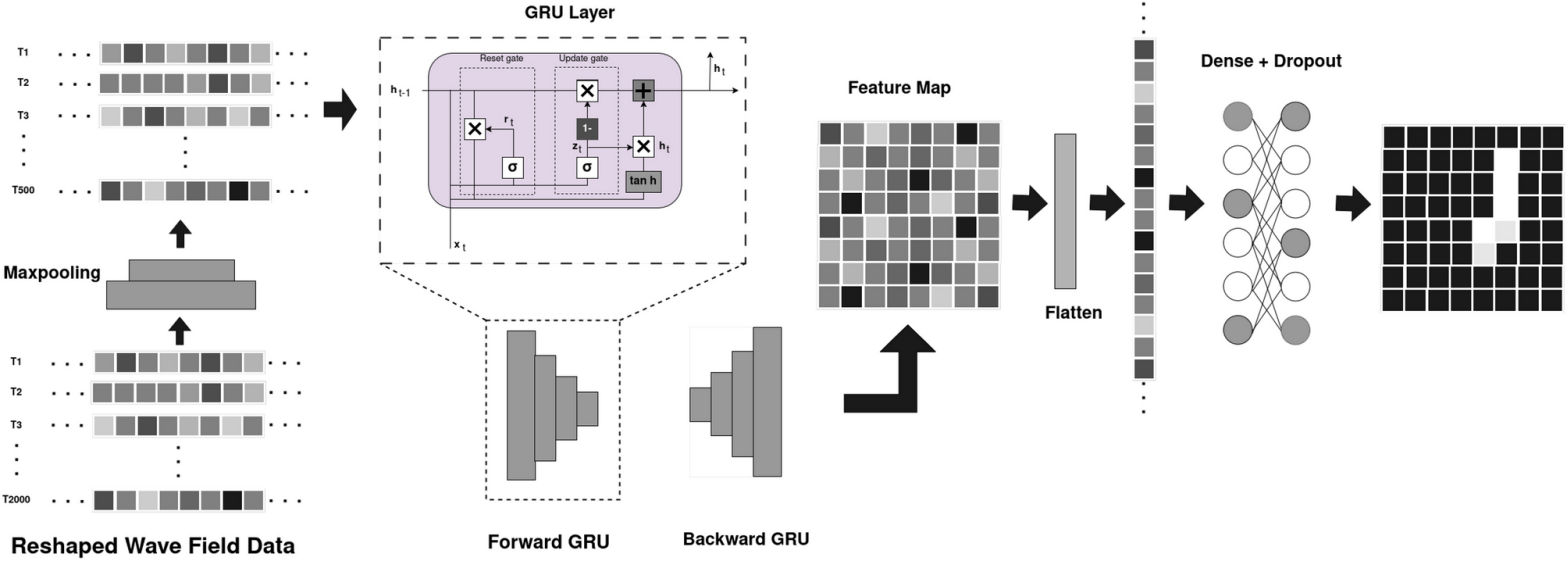
Architecture of the Deep-learning MicroCrackRNNNet Model.

**Fig. 3:**
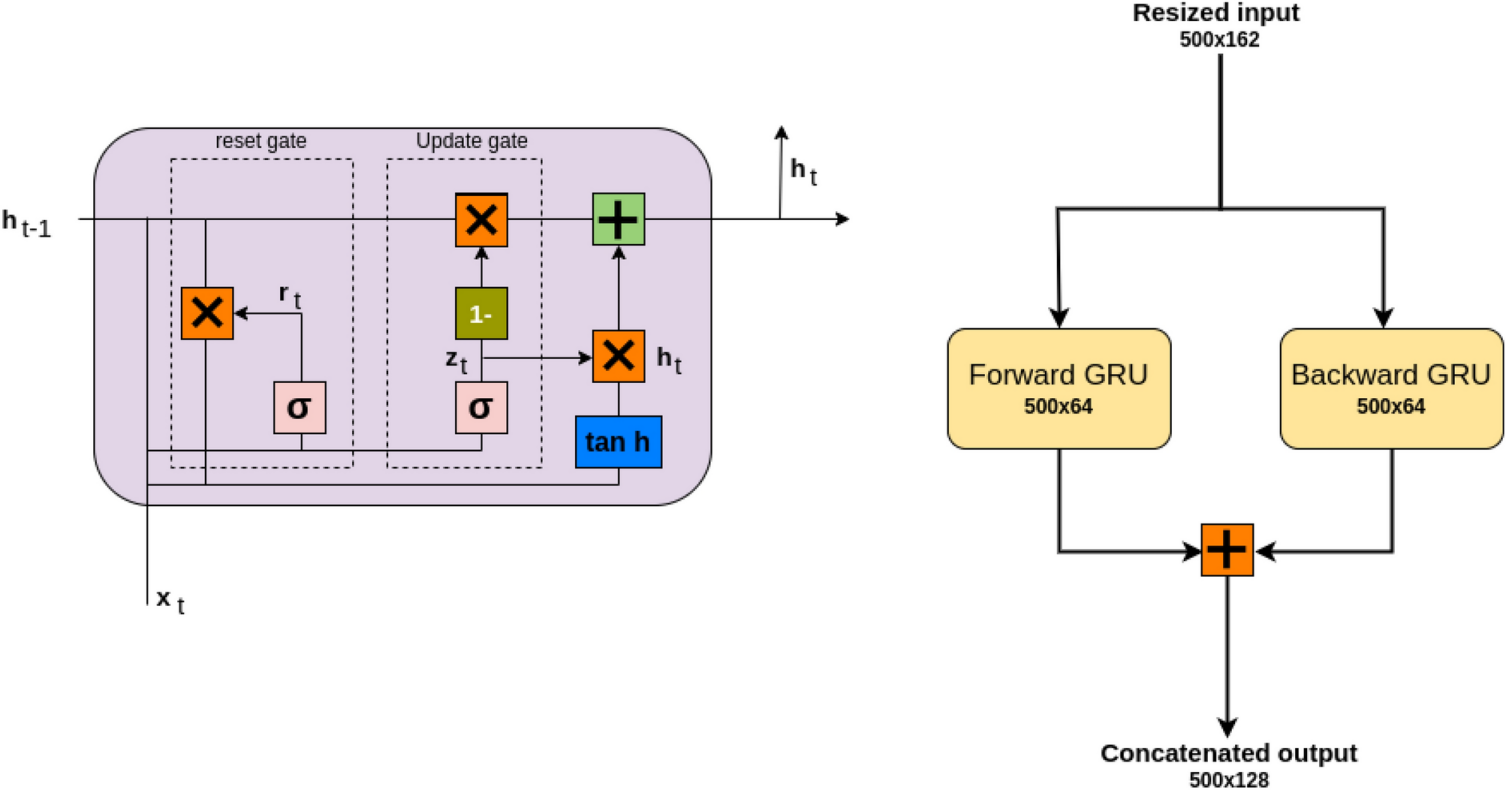
Gated Recurrent Unit and Bi-Directional GRU.

Our Problem is comparable to Sementic segmentation task, the previous work^3^ focused on using convolutional layers in a Encoder Decoder architecture and using deep neural networks (DenseNet^3^ ) to extract features from the wave filed data. In this study the model has a simplistic hybrid CNN+RNN model (Fig. 2) to extract the temporal and spatial features, it uses a single Gated Recurrent Unit (GRU) (Fig. 3) layer to extract the features from the wavefield data followed by Dense layers.

### MicroCracksRNNNet network model

As discussed in the previous sections the input data is of shape 2000x81x2 where, The spatial features are combined with the channels with the help of reshape layer, a 1D Maxpooling layer reduces the temporal features by 4 to 500 allowing only the prominent features to pass on reducing the computational requirements and discard the less prominent information from the data, allowing the model to focus on the most prominent features.

The GRU processes each time step in the sequence, learning temporal patterns by keeping track of features from previous time steps. In a Bidirectional GRU (Fig. 3), the data is passed through two GRU layers: one moving forward (from start to end of the sequence) and one moving backward (from end to start). This approach enables the model to understand the context from both directions, which can be crucial in tasks where future information might inform the interpretation of past data.

The GRU layer outputs a sequence with shape $500 \times 128$ (since it has 64 units in each direction due to the bidirectional structure, resulting in a combined output size of 128). This output is a transformed representation of the input, where each time step now encapsulates learned temporal relationships and contextual dependencies. The GRU’s sequential processing allows it to capture long-range dependencies, which is critical for detecting features that span across multiple time steps. This is especially relevant in tasks like crack detection, where the presence of a crack might be detected over a sequence of frames rather than in a single frame.

The final output from the GRU layer, which has a shape of $500 \times 128$, is flattened into a one-dimensional array. This transformation prepares the data for any subsequent dense layers or the output layer, condensing the temporal and learned feature information into a single vector that can be used for prediction or classification. The GRU layer is chosen here due to its efficiency in capturing temporal dependencies without the complexity of a full Long Short-Term Memory (LSTM) unit. GRUs are computationally lighter than LSTMs because they use fewer gates, making them faster and more efficient for processing sequences like this dataset. The bidirectional nature of the GRU further enhances its ability to learn from the entire context of the data, providing the model with both past and future insights at each time step. This makes the GRU layer well-suited for tasks involving sequential data, where understanding patterns across time is critical. A Dropout layer with a dropout rate of 0.2 follows the flatten layer to prevent overfitting. This regularization technique ensures that the model does not become overly dependent on specific features and helps improve its generalization on unseen data. This model architecture efficiently processes the input data by reducing its temporal dimension, then using a Bidirectional GRU to capture comprehensive temporal patterns, followed by dropout regularization and flattening. The GRU layer plays a pivotal role in transforming the sequence data, allowing the model to learn time-based dependencies without the need for a complex decoder, which would otherwise increase computational demands. This streamlined approach, with the GRU at its core, enables effective segmentation and feature extraction, making it suitable for tasks like microcrack detection, where temporal context is key.

### Gated recurrent unit

The Gated Recurrent Unit (GRU) is an advanced version of the recurrent neural network (RNN). Simple RNNs struggle to retain long-term sequences in time-series data, which led to the introduction of Long Short-Term Memory (LSTM) networks. LSTMs can retain both long- and short-term context using two states and control the information flow through three gates. However, due to its complex architecture with a high number of parameters, LSTMs can increase training time and computational cost. To address this, GRUs were introduced with a simpler architecture, fewer parameters, and comparable performance to LSTMs. GRUs retain both long- and short-term context with only a single hidden state, rather than using two separate states, and rely on two gates:

- Reset Gate
- Update Gate

In a GRU, at any timestamp $t$, two inputs are considered: the previous hidden state and the current input, The gates in a GRU balance and control the flow of information, allowing it to compute the current hidden state $h_t$ based on the current input $x_t$ and the previous hidden state $h_{t-1}$. This is achieved through a two-step process:

- Reset Gate ($r_t$) : This gate determines how much of the past information to forget based on the current input. The reset gate adjusts the past information by creating a weighted sum of the previous hidden state $h_{t-1}$ and the current input $x_t$, which is then passed through a sigmoid activation function to scale it between 0 and 1. The reset gate equation is:
  1 $$\begin{aligned} r_t = \sigma (W_r \cdot [h_{t-1}, x_t] + b_r) \end{aligned}$$
- Candidate Hidden State ($\tilde{h}_t$) : This intermediate vector serves as a potential hidden state. It is formed by an element-wise multiplication of $r_t$ with $h_{t-1}$, which is then processed similarly to the reset gate but with a tanh activation function instead of sigmoid:
  2 $$\begin{aligned} \tilde{h}_t = \tanh (W_c \cdot [h_{t-1} *r_t, x_t] + b_c) \end{aligned}$$
- Update Gate ($z_t$) : This gate controls how much of the past memory to retain, balancing between the previous hidden state and the candidate hidden state.
  3 $$\begin{aligned} z_t = \sigma (W_z \cdot [h_{t-1}, x_t] + b_z) \end{aligned}$$
  The final hidden state is computed by blending the previous hidden state and the candidate hidden state based on the update gate, If $z_t$ is greater than 0.5, the current input has a stronger influence on the current hidden state, and the past information is given less weight. Conversely, if $z_t$ is less than 0.5, the next state retains more influence from the previous state. The update gate equation is:
  4 $$\begin{aligned} h_t = (1 - z_t) *h_{t-1} + z_t *\tilde{h}_t \end{aligned}$$

This simplified architecture of the GRU reduces the parameter count while maintaining the ability to capture both short- and long-term dependencies in the data effectively.

### CNNs vs RNNs

Both Recurrent Neural Networks (RNNs) and Convolutional Neural Networks (CNNs) are fundamental deep learning architectures, each designed to address specific types of data and challenges. Their distinct properties make them suitable for a range of applications, with CNNs excelling in spatial analysis and RNNs in sequential data processing.

#### Convolutional neural networks (CNNs)

Convolutional Neural Networks (CNNs) are an essential class of deep learning models that use multiple layers to extract spatial features from data^22,23^, performing tasks like object detection, segmentation, and classification.

In a CNN, the layers are composed of neurons arranged in three dimensions: the height and width of the input, and the depth. The term “depth” here refers to the third dimension of the activation volume, rather than the number of layers in the network. Unlike traditional ANNs, neurons in each CNN layer are connected only to a localized region of the previous layer.

A CNN mainly comprises three types of layers: convolutional, activation function layers and pooling. Convolutional layers form the core of CNNs and consist of learnable kernels that extract features from the input data. Larger kernel sizes capture global features, while smaller kernel sizes focus on more local features. The number of training parameters increases with the kernel size. Convolutional layers can be 1D, 2D, or 3D based on kernel dimensionality, with examples like 1D (2x1, 3x1), 2D (2x2, 3x3), and 3D (2x2x2, 3x3x3) kernels. Previous studies have utilized 1D convolutional layers.

Forward Propagation (FP) and Back Propagation (BP) in 1D CNNs require simpler array operations, making 1D CNNs significantly less computationally complex than 2D CNNs. A 1D CNN extracts information from 1D signals by applying 1D convolutions, where filters (kernels) slide across the input signal array. Unlike 2D CNNs, which use 2D kernels to process 2D matrices (such as images), 1D CNNs apply 1D filters to 1D data arrays (such as time series or signals).

In each 1D CNN layer, the kernel computes a weighted sum over a segment of the 1D signal, which is then scaled by passing it via activation function. The output is subsequently sub-sampled (pooled), compressing the feature map and reducing data dimensions. This process enables the model to learn key structural and pattern features in the signal, allowing it to efficiently perform feature extraction and classification on 1D data.

Pooling layers^24^ reduce the dimensionality of the feature maps extracted from convolutional layers, thus decreasing computational complexity and the number of training parameters in subsequent layers. Pooling layers do not have training parameters; instead, they reduce dimensionality by filtering out less relevant information and passing only the most prominent features to the next stage. Two most common pooling techniques are Max pooling and average pooling: max pooling retains the maximum values from feature maps, while average pooling averages the values.

#### Recurrent neural networks (RNNs)

Time series data, characterized by sequential dependencies, requires models that capture patterns over time. RNNs are particularly suitable for this as they include feedback connections, enabling them to retain information about previous inputs over time, which is essential for tasks like language modeling, speech recognition, and predictive modeling in sequential data contexts. Unlike traditional feed-forward neural networks that process each input independently, RNNs maintain a “memory” of past inputs, allowing them to model temporal dependencies. This is crucial when predicting future events based on past patterns, that is the case with the time series data. In an RNN, each neuron in a layer can receive input not only from the preceding layer but also from the previous time step’s neurons, forming a loop within the network. This loop structure allows RNNs to maintain a hidden state that is updated at each time step, effectively carrying information across the sequence. The recurrent connections help capture dependencies in sequential data, a task that ANNs struggle with since they lack this ability to handle sequences natively.

Artificial Neural Networks (ANNs), such as standard feed-forward networks^25^, are limited in their application to static data, where each input is independent of others. They do not account for the order or context of inputs, which is essential in time-dependent tasks. For instance, in a time series forecasting scenario, ANNs would treat each time step separately, thus failing to model the progression and temporal patterns within the data. In contrast, RNNs address this by “unrolling” the network across time steps, allowing each output to depend on both current and previous inputs through recurrent connections. This makes RNNs inherently better suited to tasks that require learning from sequences. CNNs use convolutional layers to detect local patterns in data and are effective for spatial data but lack the sequential dependency feature of RNNs. CNNs might capture temporal information over short local windows but are less effective in long-range temporal dependencies, which RNNs handle through recurrent connections RNNs’ ability to maintain a temporal memory differentiates them fundamentally from ANNs and renders them effective for sequential data analysis. By utilizing the information from previous time steps, RNNs model complex time-dependent patterns, making them essential for applications involving time series data.

## Benchmarking experiments

This section outlines the benchmarking outcomes for the MicroCrackRNNNet model in the crack detection task, specifically in localizing the micro scale cracks in a sample. We begin by detailing the loss function, evaluation metrics, and hyperparameters used in our experiments.

### Activation functions

The final Dense layer uses the sigmoid function (activation=’sigmoid’)^26,27^ as its activation function. In binary classification tasks^28^, the sigmoid activation function is frequently used to estimate probabilities for two classes, 0 and 1. It interprets the result as the likelihood that the input is in the positive class by squashing the output of the preceding layer into a range between 0 and 1.

5 $$\begin{aligned} \sigma (x) = \frac{1}{1 + e^{-x}} \end{aligned}$$

- $\sigma (x)$ is the output of the sigmoid function,
- $x$ is the input to the function.

Because it yields an output that is probability-like (i.e. between 0 and 1) and can be easily deduced as the chance that the input to the function belongs to which class, the sigmoid activation function is a good fit in this situation. Because ReLU^29^ is prone to vanishing gradients, learning may be hampered in deeper networks. However, with this particular architecture, with a relatively shallow network and applied dropout for regularization, this issue is mitigated to some extent.

**Table 1:** Comparison between the accuracy of the model versus different crack sizes for different Loss functions.

Crack Size	Dice Loss	Focal Loss	Assymetric Focal Loss
${\textbf {>0}}\, \varvec{\upmu }{{\textbf {m}}}$	0.7670	0.7920	0.7784
${\textbf {>0.001}}\, \varvec{\upmu }{{\textbf {m}}}$	0.8100	0.8357	0.8210
${\textbf {>0.002}}\, \varvec{\upmu }{{\textbf {m}}}$	0.9031	0.9247	0.9092
${\textbf {>0.003}}\, \varvec{\upmu }{{\textbf {m}}}$	0.9463	0.9570	0.9447
${\textbf {>0.004}}\, \varvec{\upmu }{{\textbf {m}}}$	0.9693	0.9766	0.9648

### The loss functions

We experimented with three Loss functions (Table 1): Dice Loss^30^, Focal Loss^31^ and Asymmetric focal Loss^32^ in our study, the focal loss showed the best results. The dice loss function, which was previously described in^3^. Here, we provide a brief summary of the function for completeness.

Where $\textbf{I}= \sum _1^{N} y_i p_i$, this term is used to indicate the point when the predicted output and the ground truth overlap. It’s the total of the element-wise products of the predicted probability $(p_i)$ and the ground truth labels $(y_i)$. In essence, this gauges the degree of overlap between the prediction and the actual data, $\textbf{U}= \sum _1^{N}( y_i + p_i )$, this refers to the combination of the ground truth and expected segmentation. It is the sum of the ground truth labels plus the anticipated probability. Unlike intersection, this word refers to both the ground truth and forecast regions.and $\epsilon$ is the smooth term.

For binary classification, the Focal Loss, denoted as BinaryFocalLoss, can be defined by the equation 6:

6 $$\begin{aligned} FL(p_t) = -\alpha (1 - p_t)^{\gamma } \log (p_t) \end{aligned}$$

where $p_t$ is defined as:

7 $$\begin{aligned} p_t = {\left\{ \begin{array}{ll} p & \text {if } y_{\text {true}} = 1 \\ 1 - p & \text {otherwise} \end{array}\right. } \end{aligned}$$

and $p = \sigma (x)$ is the predicted probability. The complete equation for Focal Loss, combining both positive and negative part, is:

8 $$\begin{aligned} FL(y_{\text {true}}, y_{\text {pred}}) = -\alpha y_{\text {true}} (1 - y_{\text {pred}})^{\gamma } \log (y_{\text {pred}}) - (1 - \alpha )(1 - y_{\text {true}}) y_{\text {pred}}^{\gamma } \log (1 - y_{\text {pred}}) \end{aligned}$$

This loss function calculates the loss for the positive class as $-\alpha (1 - p_t)^{\gamma } \log (p_t)$ and for the negative class as $-(1 - \alpha ) (p_t)^{\gamma } \log (1 - p_t)$ The term $(1 - p_t)^{\gamma }$ is used to down-weight well-classified examples (where $p_t$ is close to 1). The parameter $\gamma$ controls the focusing strength, with higher values putting more emphasis on hard-to-classify examples.

In contrast, the Binary Asymmetric Focal Loss modifies the Focal Loss by applying the focusing term only to the negative class. The equation for Asymmetric Focal Loss is:

9 $$\begin{aligned} AFL(y_{\text {true}}, y_{\text {pred}}) = -\alpha y_{\text {true}} \log (y_{\text {pred}}) - (1 - \alpha )(1 - y_{\text {true}}) y_{\text {pred}}^{\gamma } \log (1 - y_{\text {pred}}) \end{aligned}$$

In this case, the loss for the positive class is $-\alpha \log (p_t)$ and the loss for the negative class is $-(1 - \alpha ) (p_t)^{\gamma } \log (1 - p_t)$ The focusing term $(1 - p_t)^{\gamma }$ is only applied to the negative class (where $y_{\text {true}} = 0$), while the positive class (where $y_{\text {true}} = 1$) does not include this term. This asymmetry means that the loss function focuses more on the negative class.

### Metrics

For the evaluation part, we utilized the same metrics as in our previous paper^3^, namely the Dice coefficient (DSC) and the Intersection over Union (IOU) metric. The IoU tells how accurate an object recognition or segmentation method is. Its definition is the area of their union divided by the overlap region between the ground truth and the predicted bounding box (also known as the segmentation mask). Perfect alignment between the expected and actual regions is denoted by a 1 on the IoU scale, which goes from 0 to 1. This measure is essential for evaluating how well models perform in tasks such as picture segmentation and object detection. The accuracy calculation is dependent on the IOU value of each prediction, if the IOU values exceeds a threshold (the IOU threshold is set to 0.5 ) value then its considered as a correct prediction.

The mathematical equations of these matrices are given bellow :

10 $$\begin{aligned} F= & \frac{2\cdot precision\cdot recall}{precision+recall} \end{aligned}$$

11 $$\begin{aligned} \textrm{IoU}= & \frac{\textrm{TP}}{\textrm{TP}+\textrm{FP}+\textrm{FN}}. \end{aligned}$$

12 $$\begin{aligned} \textrm{DSC}= & \frac{\textrm{2TP}}{\textrm{2TP}+\textrm{FP}+\textrm{FN}}. \end{aligned}$$

13 $$\begin{aligned} \textrm{Acc}= & {\left\{ \begin{array}{ll} 1 & \textrm{if } \textrm{IoU}(y_{\textrm{true}}, \hat{y}_{\textrm{pred}}) > t_{\textrm{IOU}} \\ 0 & \textrm{otherwise} \end{array}\right. } \end{aligned}$$

### Settings and hyperparameters

The MicroCracksRNNNet was trained for 50 to 200 epochs using the Adam optimizer with a default learning rate of 0.001. The optimal performance was achieved at 50 epochs, after which the metric values saturated. The model exhibiting the highest Dice Similarity Coefficient (DSC) on the test data was selected as the best model.

MicroCracksRNNNet uses a dropout layer (droupout rate: 0.8) after the Bi-directional GRU layer that helps to regularize the output and prevent over fitting. The droput layer helps to drop some random neurons and connections increasing the diversity in the model training

The hyper parameters $\gamma$ and $\alpha$ play crucial roles in these loss functions Eq:8 and 9. $\gamma$ (Focusing parameter) controls the strength of the modulating factor $(1 - p_t)^{\gamma }$. Increasing $\gamma$ puts more focus on hard-to-classify examples. Increasing the $\gamma$ makes the MicroCrackRNNNet focus more on minority class or harder-to-classify examples, which can be beneficial in imbalanced datasets. For our crack detection problem, where background pixels are more than crack pixels, increasing $\gamma$ will focus more on learning the crack pixels (the positive class), which are harder to classify, whereas, $\alpha$ (Balancing parameter) balances the importance of positive vs. negative class. $\alpha$ is a weight factor for the positive class, and 1-$\alpha$ is the weight for the negative class. Setting $\alpha$ close to 1 increases the importance of the positive class, setting a higher $\alpha$ will ensure that the model pays more attention to the crack pixels. This is useful when the positive class (cracks) is underrepresented compared to the background. In our case we experimented with different values of $\gamma$ and $\alpha$, since increasing the $\gamma$ will help to focus more on the positive class(cracks) so experiments were condusted with $\gamma$=2 and 5 and $\alpha$=0.1, 0.2, 0.2555, 0.3, 0.35, 0.5, 0.75, 0.9.

Our experiments showed that:

- With $\gamma$=0: The focal loss function gets reduced to the cross entropy loss (Fig.: 4).
- With $\gamma$=2: The model began to emphasize the harder-to-classify crack pixels more effectively. At this setting, we noticed a balanced trade-off between learning both classes and maintaining stability in training.
- With $\gamma$=5: The model focused even more on the crack pixels, but care had to be taken as overly high values of $\gamma$ might lead to instability or slower convergence due to over-focusing on a few crack pixels.$\gamma$=5, the model puts a very strong emphasis on crack pixels. This might cause the model to focus too much on a small subset of crack pixels, potentially at the expense of general performance across the entire dataset. By concentrating too much on the crack pixels, the model may neglect easy to classify pixels or noise, leading to overfitting on those crack pixels and not generalizing well.

**Fig. 4:**
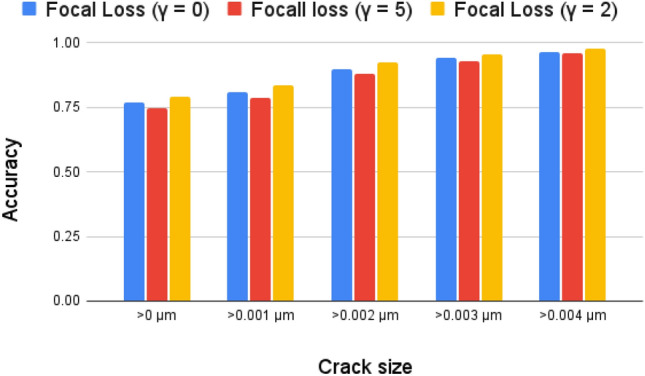
Accuracy vs. Crack Size for Model Trained with Focal Loss ($\gamma$=0,2,5).

- With varying $\alpha$ ($\gamma =2$): We observed that (Table 2) $\alpha$=0.25 to 0.5 provided a good balance, as it adjusted the focus toward the positive class without neglecting the background. Higher values like $\alpha$=0.75 or 0.9 made the model focus heavily on cracks, which was beneficial when cracks were extremely rare but could lead to overfitting and thus lower precision (Table 2) on testing data i.e unseen data.

**Table 2:** The evaluation results trained by varying $\alpha$ for Focal loss($\gamma$ = 2, dropout rate 0.2).

alpha	precision	recall	accuracy	iou	dsc
0.1	0.9257	0.64367	0.7585	0.6007	0.6934
0.2	0.9235	0.6585	0.7682	0.6107	0.7026
0.2555	0.9016	0.6743	0.7625	0.6179	0.7119
0.3	0.8955	0.6799	0.746	0.6257	0.7186
0.35	0.8947	0.6747	0.7858	0.6209	0.7138
0.5	0.909	0.6588	0.7818	0.6095	0.7032
0.75	0.8113	0.7266	0.7852	0.631	0.7284
0.9	0.8654	0.7022	0.7886	0.6253	0.7188

These experiments helped us fine-tune the model’s sensitivity towards crack detection, improving accuracy and generalization on our dataset

## MicroCracksRNNNet results

The problem discussed in this study is comparable with the segmentation problem, the numerical wavefield data that captures the information about the interaction of seismic waves with the crack is used to generate the binary maps of the cracks for localizing, visualizing the micro-cracks providing a non invasive method solution the the problem of crack detection.

The MicroCracksRNNNet model, leveraging a Gated Recurrent Unit (GRU) for processing numerical data, demonstrates superior performance in crack detection. Precision, which represents the ratio of true positive predictions to all positive predictions, showcases MicroCracksRNNNet’s ability to minimize false positives. Similarly, recall, which indicates the capability to detect a larger proportion of actual positive instances, is high in MicroCracksRNNNet, highlighting its better sensitivity in identifying cracks. Moreover, the Intersection over Union (IoU) metric, which evaluates the overlap between predicted and actual crack regions, is significantly greater for the MicroCracksRNNNet.

The model’s performance over different Loss functions is shown in Table 1 to address different crack sizes. The focal loss function works best for the MicroCracksRNNNet, particularly for larger crack sizes, achieving an accuracy of 97%. The accuracy decreases with the crack size since the deflection of the seismic waves decreases with smaller cracks making it hard to localize the cracks.

**Fig. 5:**
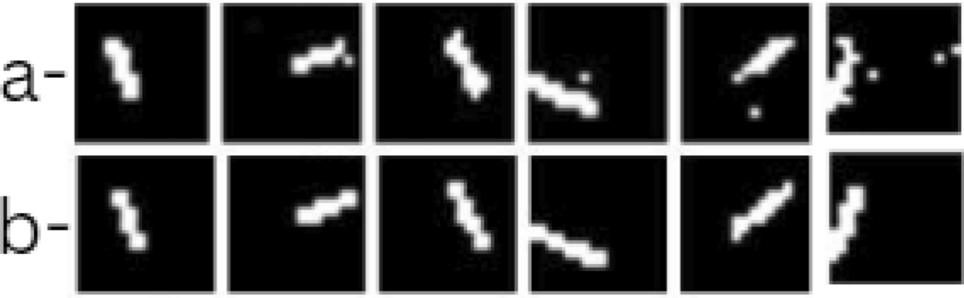
Model (**a**) predictions in comparison to (**b**) ground truths for large cracks.

**Fig. 6:**
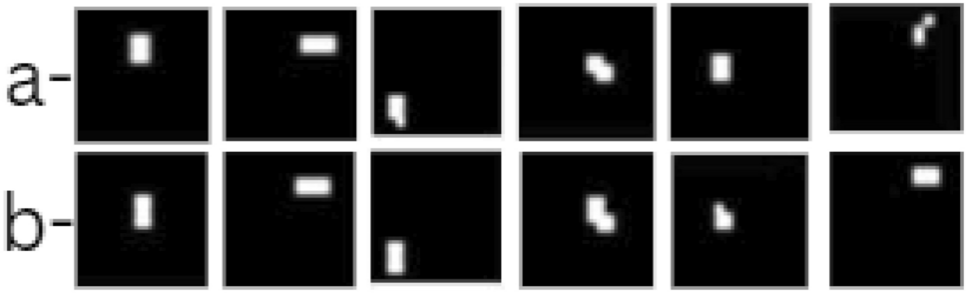
Model (**a**) predictions in comparison to (**b**) ground truths for small cracks.

**Fig. 7:**
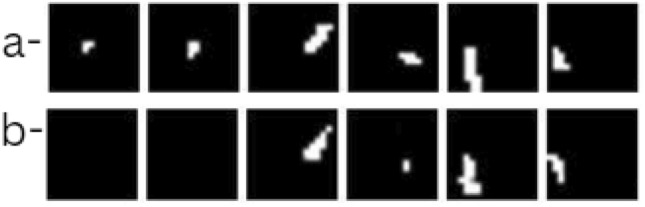
Model (**a**) predictions in comparison to (**b**) ground truths.

In Fig. 5, the model performs well on larger cracks. The predicted crack regions (row a) show a high overlap with the ground truth (row b), demonstrating the model’s strong ability to capture and localize cracks. The high accuracy with larger cracks is consistent with the model’s performance metrics, as the seismic wave deflection is more pronounced for larger cracks, aiding detection. The Fig. 6 shows model performance on smaller cracks. Although the model predictions align with the ground truth to some extent, the performance begins to drop slightly. This is visible in minor discrepancies between the predicted and actual crack locations, where the model misses or partially identifies certain crack sections. Smaller crack sizes reduce seismic wave deflection, making accurate localization challenging and reflecting the decrease in detection accuracy. In the Fig. 7, the model struggles significantly, showing a poor overlap between predicted cracks and ground truth. These cases likely represent the smallest and faintest cracks where the model fails to capture the crack regions accurately, either due to very weak wave deflection or noise interference. This set illustrates the model’s limitations, emphasizing the difficulty in identifying minute crack details.

This visual evaluation across different crack sizes highlights the MicroCracksRNNNet’s strengths with larger cracks and its challenges with smaller and less detectable cracks, supporting the numerical results discussed in Table 1.

The development of the MicroCrackRNNNet architecture involved systematic experimentation with different variants (Table 3), each tailored to evaluate specific design choices. The table provides a comparative overview of their performance across various crack size thresholds. Below we discuss the design choices and their impact on the model’s accuracy, focusing on how the MicroCrackRNNNet achieves superior results.

- MicroCrackRNNNet-D256 This variant introduced an additional dense layer with 256 output features to test whether increasing the model’s capacity at the fully connected stage could improve feature representation. It showed moderate performance improvements over a baseline (Table 3) but failed to generalize effectively for smaller cracks ($>0\, \upmu$m and $>0.001\, \upmu$m) compared to the finalized MicroCrackRNNNet. This suggests that the increase in capacity at this stage was not sufficient to optimally capture the complexity of smaller crack features.
- MicroCrackRNNNet-D256x2 Building on the previous variant, this design added two dense layers with 256 output features each. The aim was to assess the benefits of a fully connected and deeper network. Although the additional layer increased depth, the model struggled with overfitting or diminishing returns in feature abstraction. Its accuracy lags behind both the D256 variant and the MicroCrackRNNNet across all crack sizes.
- MicroCrackRNNNet-D128+256 This variant introduced a sequential dense layer design, starting with 128 output channels followed by 256 channels. The intention was to evaluate whether a gradual increase in feature dimensionality could enhance learning. This gradual increase in feature size provided some improvements in accuracy, but remained suboptimal. The model did not consistently outperform the D256 variant or the finalized architecture, suggesting that the sequential increase did not align well with the hierarchical feature requirements.
- MicroCrackRNNNet-MP1/8 In this variant, the initial max-pooling layer was modified to reduce temporal features by a factor of 8 instead of 4. This design aimed to achieve greater compression of the input data and reduce computational requirements further. Although this variant improved computational efficiency, it compromised critical temporal details, especially for finer-grained crack detection tasks. This led to reduced accuracy for smaller cracks ($>0\, \upmu$m, $>0.001\, \upmu$m), even though performance for larger cracks was relatively competitive.
- MicroCrackRNNNet-MP1/8 D256x2 This variant combined the MP1/8 and D256x2 architectures, incorporating a more aggressive max-pooling layer (1/8) and two dense layers with 256 output features each. However, performance suffered, particularly for small crack sizes, where it achieved 0.7233 at $>0\, \upmu$m-one of the lowest among all variants. This suggests that excessive compression coupled with deeper fully connected layers negatively impacted generalization.
- MicroCrackRNNNet-2xGRU This variant introduced an additional GRU layer with the same output features as the original. Furthermore, it was trained for more epochs, but the results showed saturation after 70 epochs, indicating that prolonged training did not significantly improve performance. Although it outperformed some variants (0.7364 at $>0\, \upmu$m), it was still notably weaker than the finalized MicroCrackRNNNet (0.7886 at $>0\, \upmu$m), suggesting that adding more recurrent layers did not necessarily enhance feature extraction.

**Table 3:** Comparison between the accuracy of the model versus different crack sizes for different MicroCrackRNNNet variants.

Models	Crack Size				
	${\textbf {>0}}\, \varvec{\upmu }{{\textbf {m}}}$	${\textbf {>0.001}}\, \varvec{\upmu }{{\textbf {m}}}$	${\textbf {>0.002}}\, \varvec{\upmu }{{\textbf {m}}}$	${\textbf {>0.003}}\, \varvec{\upmu }{{\textbf {m}}}$	${\textbf {>0.004}}\, \varvec{\upmu }{{\textbf {m}}}$
**MicroCrackRNNNet-D256**	0.7761	0.8189	0.9037	0.9371	0.9540
**MicroCrackRNNNet-MP1/8 D256x2**	0.7233	0.7626	0.8502	0.8987	0.9262
**MicroCrackRNNNet-D128+256**	0.7330	0.7734	0.8631	0.9010	0.9279
**MicroCrackRNNNet-MP1/8**	0.7619	0.8040	0.8963	0.9432	0.9657
**MicroCrackRNNNet**	0.7886	0.8321	0.9153	0.9509	0.9666
**MicroCrackRNNNet-D256x2**	0.6108	0.6445	0.7193	0.7667	0.7953
**MicroCrackRNNNet-2xGRU**	0.7364	0.7770	0.8698	0.9225	0.9513

Key Observations:

- Importance of Temporal Details: The MP1/8 variant’s performance drop underscores the importance of retaining sufficient temporal resolution in early layers.
- Feature Representation Depth: While increasing depth (D256x2) or sequential dimensionality (D128+256) did not improve results, a carefully calibrated depth in the finalized MicroCrackRNNNet strikes an optimal balance.
- Generalization Across Scales: The finalized architecture consistently outperforms variants for all crack sizes, indicating robust feature learning and generalization.

The MicroCrackRNNNet architecture outshines its experimental variants by maintaining a thoughtful balance between computational efficiency, model capacity, and feature resolution. These results highlight the critical role of architectural design in addressing fine-grained tasks like microcrack detection, where small-scale details heavily influence overall performance.

## Beyond the encoder-decoder paradigm

The traditional encoder-decoder architecture^33^ has long been favored for image segmentation tasks due to its ability to capture local and global information hierarchically. By encoding complex input data into a compressed representation and then decoding it back to its original size, this approach has found widespread use in fields such as medical imaging, satellite imagery, and object detection. However, despite its advantages, the encoder-decoder architecture presents significant limitations, particularly due to the heavy computational cost and high parameter count in the decoder stage. These requirements make the model prone to overfitting, require time-intensive tuning, and limit its applicability in resource-constrained environments, such as mobile and embedded systems.

To address these challenges, this work explores RNN-based models, specifically those using bidirectional Gated Recurrent Units (GRUs), as a more efficient alternative for segmentation tasks. Unlike encoder-decoder models, RNNs do not rely on an extensive decoding process. Instead, they process data sequentially, allowing them to inherently capture temporal dependencies within data. This characteristic is particularly advantageous for crack detection, where temporal patterns in seismic or wavefield data can reveal subtle, evolving cracks. By focusing on temporal connections, RNNs excel at tracking such progression over time, which conventional convolutional architectures may struggle to capture without extensive modifications.

The simplicity of RNNs provides additional benefits. Although RNNs have a relatively high parameter count, they use fewer layers than typical encoder-decoder models, resulting in a streamlined architecture that reduces the risk of overfitting. This compact design is computationally less demanding, making RNNs suitable for deployment on edge devices and low-power platforms. As a result, our RNN-based model supports real-time inference, an essential feature in applications like predictive maintenance, where quick processing is crucial. Moreover, RNNs can be enhanced with attention mechanisms, allowing the model to focus selectively on relevant parts of the sequence, further improving accuracy without adding significant computational overhead.

In conclusion, RNN-based model offers a competitive, resource-efficient alternative to traditional encoder-decoder architectures for segmentation tasks. By eliminating the need for a complex decoder, it minimizes computational demands and reduces latency, making it ideal for environments with limited processing power. Furthermore, the ability to capture temporal dependencies positions RNNs as particularly effective in tasks that require sequential pattern recognition, such as crack detection. Our findings underscore the potential of RNNs to broaden the applicability of segmentation models beyond the limitations of encoder-decoder structures, paving the way for advancements in real-time and resource-sensitive applications.

**Table 4:** Comparitive analysis based on parameters and training time.

Attributes	MicroCracksRNNNet	1D-DenseNet50E	1D-DenseNet200E
Layers	8	444	444
Epochs	50	50	200
Time taken by first Epoch	42.6600 sec	89.1400 sec	89.14 sec
Total training time	1,782.1600 sec	3,890.1400 sec	15,560.5600 sec
Total params	16,471,808	1,393,429	1,393,429
Trainable params	16,471,808	1,376,137	1,376,137
Non-trainable params	0	17,292	17,292
Accuracy	0.7890	0.8120	0.8360
Precision	0.9260	0.7220	0.8750
Recall	0.7270	0.6940	0.8520

The table presents a comparative analysis of the Recurrent Neural Network (RNN) model against two 1D-DenseNet variants, focusing on various attributes, including the number of layers, epochs, training time, and parameters.

RNN Model Overview:

- Layers: The RNN model consists of only 8 layers (Table 4), significantly fewer than the 444 layers in both DenseNet variants. This simplicity in architecture can be an advantage when dealing with smaller datasets or tasks requiring less computational complexity.
- Training Time: Despite its simplicity, the RNN model required a total training time of approximately 1782.1600 seconds (Table 4). This is faster compared to both DenseNet variants, especially the DenseNet200E, which required over 15,560 seconds (Table 4). The RNN’s shorter training time might be advantageous in scenarios where quick model deployment is essential.
- Parameters: Interestingly, the RNN model has significantly more parameters (16,471,808) (Table 4) compared to the DenseNet variants (approximately 1.39 million). Initially, this might suggest a greater capacity to learn complex patterns. However, the large number of parameters could be a result of the fewer layers in the RNN model, leading to a more substantial parameter count per layer. This high parameter count increases the risk of overfitting, particularly with smaller datasets. The large parameter count in the RNN model highlights an area for potential improvement. Introducing additional max-pooling layers before the RNN layers could reduce the input dimensionality, leading to faster processing and a lower resolution. This approach would help reduce the number of parameters, potentially making the RNN model more efficient while maintaining or improving its performance. This adjustment could allow the RNN to leverage its strength in handling sequential data while mitigating the risk of overfitting.
- Trainable vs. Non-trainable Parameters: All parameters in the RNN model are trainable, which could contribute to its ability to adapt during training. However, this also means the model requires careful tuning to avoid overfitting.

The primary distinction between this study and previous work^3^ lies in the architecture of the deep learning (DL) model. In previous studies, CNN-based architectures were used, which proved to be highly effective for processing the wave field data with both temporal and spatial dimensions. The CNN encoder was responsible for extracting the most important features from the data, while the decoder reconstructed these features into the desired output shape, ensuring that the spatial structure was preserved. This architecture was instrumental in addressing the dual temporal and spatial nature of wavefield data. However, in this study, a completely new approach is used using RNN units, eliminating both the decoder and the exclusive dependence on CNNs. Our approach removes the decoder entirely while still achieving strong performance. This reduction is particularly advantageous, as it significantly lowers the complexity of the model. The number of layers has been drastically reduced, resulting in a much more efficient architecture. Instead of relying on a full decoder, the features extracted by the encoder are directly processed by a dense layer, which generates the final output. Our results demonstrate that this simplified approach is not only feasible but also achieves comparable performance, leading to a substantial improvement in efficiency. Another key innovation in this work is the introduction of a hybrid architecture that combines components from CNNs and RNNs. This marks a significant departure from our previous work, which exclusively employed CNNs. The hybrid architecture enables us to take the advantage of the strengths of both approaches. CNNs, which are traditionally specialized in extracting spatial features, and RNNs, which excel at modeling temporal dependencies. However, our analysis shows that this division is not strictly adhered to in practice. The bidirectional GRU layer (RNN) in our model plays a central role, effectively processing both spatial and temporal dependencies. At the same time, CNN components contribute by capturing patterns that are relevant to both dimensions. This synergy makes the hybrid architecture particularly well-suited for wavefield data.

The lower accuracy of 78.9% compared to traditional CNN model^3^ can be justified by the benefits of the new solution in terms of computational efficiency and simplicity. Despite a higher number of trainable parameters, the model achieves this with significantly fewer layers, resulting in faster training times and better memory efficiency. This is because the 444-layer model introduces a lot of overhead due to its depth, despite having fewer parameters overall. The trade-off in accuracy is acceptable, as the approach offers a more practical solution in scenarios where speed and resource constraints are crucial. Overall, the advantages of faster processing and reduced complexity outweigh the slight drop in accuracy, opening up new opportunities for further optimization in real-world applications.

## Future work

This work presents a simple model structure comprising RNN and CNN layers. The primary goal of this study was to demonstrate that this combination could be advantageous. The results show that by using a single RNN layer-specifically, a GRU layer-significant performance improvements can be achieved. This finding motivates the development of more complex models where RNNs play a more prominent role. One limitation of the current work is that the data is in 2D, whereas a 3D data would be more realistic, as it would better reflect real-world conditions. In addition, the crack shapes could be made more complex and the number of cracks per sample could be increased. In reality, multiple cracks often appear on a surface, and increasing the number of cracks in the model would prevent the model from making biased assumptions, such as one crack per sample.

Future work could explore deeper networks such as Densenet Blocks with RNN layers, especially with multiple GRU layers, to achieve better results. At the same time, more powerful CNN layers, such as additional convolutional layers, could provide an advantage. So far, we have only used CNN pooling layers; expanding the model to include more convolutional operations could further enhance its performance. Future work could focus on shifting the model’s reliance more toward RNN layers and thoroughly analyzing the results, instead of depending primarily on CNN components. Another promising direction would be to modify how data is processed by the model. A potentially interesting model could process the temporal dimension exclusively using RNNs, while using CNNs for the spatial dimension.

Additionally, integrating self-attention mechanisms would be an intriguing avenue for exploration, as RNNs combined with attention often yield successful outcomes. Currently, we are working intensively on generating more data and plan to test our models with larger datasets. This would help cover more real-world scenarios and train more robust models that can handle various edge cases.

## Summary

This study demonstrated that the combination of RNN and CNN layers delivers promising results, comparable to those of our previous complex deep models. The results suggest that this model combination could also be evaluated in other areas where both temporal and spatial dimensions play a role. The use of additional RNN layers, particularly GRU layers, could offer further benefits.

An important contribution of this study is that the hybrid approach, although not currently superior in terms of accuracy, offers new perspectives and provides opportunities to overcome existing limitations or refine the approach further.

Particularly notable is the reduction in the number of layers from 444 to just 8, representing a significant simplification without a major loss in performance. A key aspect of this approach is that it eliminates convolutional layers from the CNN family, which are typically considered essential. Instead, the model relies on recurrent layers (RNNs), which better capture the temporal aspect of the data. This focus on RNNs enables the model to achieve strong results with a much simpler architecture, when compared to previous, more complex models that involved many convolutional layers.

Another interesting feature of this hybrid model is that, despite the larger number of trainable parameters, the training time is significantly shorter. This shows that larger models do not necessarily require longer training times and opens up new possibilities for efficiently scaling models, which is of great interest for future research. Overall, this model demonstrates that by reducing complexity and focusing on recurrent layers, an efficient, robust, and scalable system can be developed that can still deliver excellent results in real-world applications, such as crack detection.

## Data Availability

The code and the datasets generated during and/or analysed during the current study are available from Fatahlla Moreh (corresponding author) on reasonable request.
